# Prevalence of and Factors Associated with Hypertension Among Bank Employees: A Cross-Sectional Study in Saudi Arabia

**DOI:** 10.3390/healthcare13020134

**Published:** 2025-01-13

**Authors:** Manal A. Al-Batanony, Bader S. Alharbi, Meshal S. Alharbi, Oqab A. Alharbi, Abdullah A. Almutairi, Mohammad F. Almansour, Osama Al-Wutayd

**Affiliations:** 1 Department of Family and Community Medicine, College of Medicine, Qassim University, Buraydah 52571, Saudi Arabia; o.alwutayd@qu.edu.sa; 2 Department of Public Health and Community Medicine, Menoufia Faculty of Medicine, Menoufia University, Shibin el Kom 6131567, Egypt; 3 College of Medicine, Qassim University, Buraydah 52571, Saudi Arabia; 391110185@qu.edu.sa (B.S.A.); 391108006@qu.edu.sa (M.S.A.); 391108248@qu.edu.sa (O.A.A.); 391110129@qu.edu.sa (A.A.A.); 391108061@qu.edu.sa (M.F.A.)

**Keywords:** hypertension, prevalence, risk factors, occupational risk, bankers, Saudi Arabia

## Abstract

**Background and Objectives**: Hypertension (HTN) is one of the most common non-communicable medical conditions and the leading preventable risk factor for early mortality worldwide. As a result of their exposure to sedentary work and job strain, bank employees comprise an occupational group at risk for HTN. Due to the lack of previous research addressing this issue in Saudi Arabia, this study aimed to assess the prevalence of HTN and its associated factors among bankers in the Qassim region, Saudi Arabia. **Materials and Methods**: This cross-sectional study was conducted among 342 bank employees. A self-administered questionnaire in the workplace was used to collect data on the sociodemographic characteristics and risk factors of participants, including smoking, physical activity, family history of HTN, and diabetes mellitus. Each participant’s blood pressure, height, and weight were measured. Cohen’s Perceived Stress Scale was used to assess stress levels. **Results**: The prevalence of HTN in the participants was 28.9%. Multiple logistic regression analysis showed that being a smoker (adjusted odds ratio [aOR] = 1.82, 95% confidence interval [CI]: 1.02–3.25), diabetic (aOR = 5.14, 95% CI: 1.60–16.54), or obese (aOR = 5.49, 95% CI: 2.75–10.96); having a positive family history of HTN (aOR = 2.48, 95% CI: 1.36–4.51); and having a very high stress score (≥21; aOR = 3.24, 95% CI: 1.04–10.11) were associated with an increased risk of HTN, while walking for 10 min continuously ≥7 times/week (aOR = 0.28, 95% CI: 0.12–0.64) was associated with a decreased risk of HTN. **Conclusions**: The findings revealed that almost one out of three bank employees had HTN. Periodic screening for early detection of HTN, as well as implementing health education and lifestyle modification programs, is recommended.

## 1. Introduction

Globally, hypertension (HTN) is considered as one of the most prevalent non-communicable diseases [1]. Approximately one billion people worldwide suffer from HTN, and its prevalence is expected to rise to 1.5 billion by 2025 [2]. As 49% of ischemic heart diseases, 62% of cardiovascular diseases, 45% of fatalities due to ischemic heart diseases, and 51% of deaths due to cerebrovascular diseases are caused by hypertension, it is considered a prominent risk factor for many heart disease-related morbidities and mortalities [3]. HTN can progress into complicated conditions without symptoms. Due to the nature of the disease, HTN is often detected after the development of cardiovascular complications, especially in developing nations where individuals present minimal health-seeking behavior for routine checkups. Thus, regular screening is required for early detection and avoiding the complicated outcomes [4].

Research has identified risk factors contributing to HTN, including older age [5,6], male sex [5,7,8], overweight/obesity [6,9], physical inactivity [6,10], smoking and alcohol use [7], unhealthy dietary habits [11,12], sedentary lifestyle and mental stress [13], family history, and diabetes mellitus (DM) [14,15].

Banking careers remain popular among job seekers due to the competitive salaries, employment security, and financial benefits offered by such positions. High levels of workplace strain, defined as a combination of high job demands and low job control, as well as a high effort–reward work imbalance, have been linked to significantly higher blood pressure (BP) [16,17]. Bankers may be more prone to high BP due to their exposure to job strain, extended working hours, and heavy workloads [18].

In Saudi Arabia, the prevalence of HTN in the general population of Riyadh City was reported as 15% for men versus 19.5% for women in 2021 (*p* = 0.003) [19]. In this regard, although bank employees constitute a group vulnerable to HTN, its prevalence had not been previously reported in any Saudi research. Thus, this study aimed to assess the prevalence of HTN and its associated factors among bank employees in the Qassim region, Saudi Arabia. This study’s results could be valuable in filling the information gap concerning the prevalence of HTN among bank employees in the Qassim region, allowing for the development of preventive interventions to improve their well-being.

## 2. Materials and Methods

### 2.1. Study Design and Sampling

A cross-sectional study was carried out among employees of commercial banks in three governorates in the Qassim region. A multi-stage random sampling technique was applied to recruit the study participants. Out of 13 governorates in the Qassim region, 3 were chosen by means of a simple random sampling technique; 7 banks from each of the selected governorates were then picked using the same sampling technique. A list of employees was taken from the bank manager, and the available employees in the list were approached by the data collectors directly to participate in the study between June and August 2024. All male and female employees of the selected banks—including managerial, official, and clerical staff—who had spent at least one year on their job were included in the study, while pregnant women were excluded from the study.

### 2.2. Sample Size Calculation

The online open-source epidemiologic statistics for public health (open *EPI*) program [20] was used to calculate the sample size. Based on a 95% confidence range, a 5% margin of error, and a 69.5% prevalence rate, as reported in a previous study [6], the estimated sample size was 310. After considering a 10% non-response rate, the sample size was adjusted to 340.

### 2.3. Survey Tool and Data Collection

The aim of this research was clearly explained to all participants before they joined the study. In the workplace, data were collected via a self-administered questionnaire—filled within 10 min by each employee—which included questions about the following data:

- Sociodemographic characteristics such as age group (in years), sex, educational level, smoking habits, and marital status;
- Questions about HTN-associated risk factors, according to the World Health Organization (WHO) STEPS Instrument [21]. This instrument is a simple standardized approach for gathering, evaluating, and sharing information on the key risk factors for non-communicable diseases across nations. Moreover, the instrument covers key biological risk factors, such as overweight and obesity, as well as behavioral risk factors such as smoking, unhealthy diet, and physical inactivity. The questionnaire was tested on a sample of 20 participants to estimate the clarity and ease of understanding of the questions, as well as the time needed for completion (results not included in the study).

### 2.4. Weight and Height Measurements

Anthropometric measurements were taken, including height and weight, in order to calculate the body mass index (BMI). The participants’ weights were measured in kilograms using an Eufy digital scale, with an error of ±0.1 kg and measured up to 180 kg. The device was placed on a solid, flat surface, with subjects standing face forward and arms at their sides with minimal clothing and no footwear. The participants’ heights were measured using a non-stretchable tape meter (SALUX Double Scale Measuring Tape—3 m) with 0.1 cm accuracy and the conventional procedures of standing without a jacket and barefoot against a wall. Prior to data collection, the weight and height devices were calibrated.

### 2.5. Blood Pressure Measurement

A mercury sphygmomanometer (KBM, Kenzmedico Co., Ltd, Japan) with proper cuff size was used by trained professionals to take each participant’s BP. After five minutes of rest, the BP was measured while the bank worker was seated, without smoking or drinking coffee or tea for at least half an hour before, with feet relaxed on the floor and arms supported at chest level. During a single visit, each participant’s BP was measured twice, separated by at least three minutes, and the mean of the two readings was used to calculate the BP. Based on the Saudi Ministry of Health [22], BP (measured in mmHg) was classified into normal BP: SBP = 120–129 and DBP = 80–84; pre-HTN BP: SBP = 130–139 and DBP = 85–89; and HTN: SBP ≥ 140 and/or DBP ≥ 90. Hypertension was considered when SBP ≥ 140, DBP ≥ 90, and/or the person used antihypertensive medications.

### 2.6. Stress Level Measurement

The validated Arabic version of Cohen’s Perceived Stress Scale (PSS-10) [23] was used to assess the bank employees’ stress levels. The PSS-10 asks participants to score how much they felt that events in their lives over the previous month were stressful, overwhelming, unpredictable, and out of control. A 5-point Likert scale was used for the responses (0 = never, 1 = rarely, 2 = sometimes, 3 = often, and 4 = always). Except for items 4, 5, 7, and 8, which are positively phrased, all other items on the PSS-10 are negatively expressed. After reverse-coding the positively worded items, the sum of all item scores yields the PPS-10’s overall score. Higher perceived stress levels are indicated by higher scores, based on the following ranges: 0–11 = low, 12–15 = average, 16–20 = high, and ≥21 = very high.

### 2.7. Data Analysis

Standard software—namely, Statistical Package of Social Sciences (SPSS) version 24.0—was used for data coding and tabulation. Numbers and percentages (n and %) were used to identify qualitative data, where the chi-square test was performed to compare between categorical data, which could contain two or more groups. Multiple logistic regression was applied to identify associated factors (e.g., age, marital status, smoking habit, family history of HTN, DM, BMI, and perceived stress level) as independent variables and HTN status as the dependent variable. Variables with a univariate *p*-value of ≤0.20 were included in the multiple logistic regression model. The crude odds ratio (cOR), adjusted odds ratio (aOR), and 95% confidence interval (CI) are reported. The significance level was set at *p* < 0.05.

### 2.8. Ethical Considerations

The Qassim Region Research Ethics Committee approved and guaranteed this work (N# 8272/45/607 on 25 December 2023). Informed consent was obtained from each bank employee after they were informed about the study’s aim (see Supplementary Material).

## 3. Results

### 3.1. Sociodemographic Characteristics

The number of bank employees working in the Qassim region who agreed to participate in this study was 342/351. The group’s sociodemographic characteristics were as follows: male (59.6%), age group younger than 39 years (74.8%), married (62.6%), with bachelor’s level of education (65.2%), smokers (34.8%), living in their own houses (67.5%), and living with less than five family members (56.7%). In the univariate analysis, with increasing age, the prevalence of HTN significantly rose (from 20.7% in the group belonging to the 20–29 age range to 66.7% in the group aged 50 years or older; *p* = 0.01). Other sociodemographic factors, including sex, marital status, educational level, the number of family members in the household, and housing type, showed non-significant differences between hypertensive and non-hypertensive bank workers (*p* > 0.05; Table 1).

### 3.2. Prevalence of Hypertension (HTN)

The HTN prevalence rate in the studied group was 28.9% (99 participants), while pre-HTN accounted for 16.4% (56 subjects), and normotensive persons represented 54.7% (187 individuals) (Table 1).

### 3.3. Studied Risk Factors for HTN

The univariate analysis revealed that factors significantly associated with HTN were obesity (55.2%), diabetes mellitus (65.2%), positive family history of HTN (37.8%), smoking (36.1%), and having high/very high perceived stress levels (28.6%/37.6%) (*p* < 0.001 for the first three and *p* = 0.03 for the last two, respectively). Bankers who walked for 10 min continually ≥7 times/week (16.5%) showed a significantly lower prevalence of HTN than those who did not (*p* = 0.005). In contrast, drinking coffee, eating vegetables and fruits, consuming fatty food, adding salt while eating food, and doing moderate physical exercise were non-significantly associated with HTN (*p* > 0.05 for all; Table 2).

### 3.4. Simple and Multiple Logistic Regression Analysis of Factors Associated with HTN Among Bank Employees

In the simple logistic regression, the age groups 30–40 years (cOR = 2.07, 95% CI: 1.03–4.16) and ≥50 years (cOR = 7.67, 95% CI: 1.75–33.67), smoking (cOR = 1.69, 95% CI: 1.04–2.73), having a positive family history of HTN (cOR = 2.94, 95% CI:1.75–4.93), diabetes (cOR = 5.25, 95% CI: 2.15–12.82), obesity (cOR = 5.78, 95% CI: 3.12–10.71), and having a very high perceived stress level (cOR = 3.52, 95% CI: 1.36–9.08) were associated with increased risk of HTN. On the other hand, walking for 10 min continuously for ≥7 times/week (cOR = 0.41, 95% CI: 0.21–0.78) was associated with decreased risk of HTN. Furthermore, the multiple logistic regression showed that smoking (aOR = 1.82, 95% CI: 1.02–3.25), diabetes (aOD = 5.14, 95% CI: 1.60–16.54), positive family history of HTN (aOR = 2.48, 95% CI: 1.36–4.51), obesity (aOR = 5.49, 95% CI: 2.75–10.96), and having very high perceived stress levels (aOR = 3.24, 95% CI: 1.04–10.11) were associated with increased risk of HTN, while walking for 10 min continuously for ≥7 times/week (aOR = 0.28, 95% CI: 0.12–0.64) was associated with decreased risk of HTN (Table 3).

## 4. Discussion

To the best of our knowledge, this is the first study to focus on the topic under investigation, not only in the Qassim region but also in Saudi Arabia. The main finding in this research was that almost one out of three bank employees had HTN. According to a recent systematic review in Saudi Arabia [24], the pooled HTN prevalence in the general population (aged 14–100 years) was 22.7%. Bank employees are not exempt from this high prevalence; the finding that about one-third of the studied bank workers were hypertensive indicated a higher rate in this group than in the general population. Although recent studies have reported a higher prevalence of HTN among bank employees in Ethiopia (52.4%) [25] and India (44.3%) [26], other research noted lower prevalence, such as in Nigeria (12.4%) [27], Nepal (11.3%) [28], Bangladesh (24.4%) [29], and another study in Ethiopia (24.8%) [30]. This discrepancy in prevalence rates might be attributed to the differences in the studied groups’ sociodemographic characteristics, especially considering the significant impact of behavioral factors on HTN. Another explanation for the observed disparity could be the varying criteria and guidelines used to identify HTN in those studies.

The multivariable analysis performed in this study revealed a significant association between smoking and HTN among bankers. Earlier research obtained similar findings [6,26,27,31]. In line with the results reported in the previous literature, a higher likelihood of having HTN was observed among bankers with a positive family history of the condition [5,7,25]. The reason could be the genetic predisposition to HTN that parents can pass on to their children. Again, the odds of having HTN increased among those who were diabetics versus non-diabetics, as has also been claimed in other studies [6,28,32].

In the surveyed group of bank workers, the respondents who were obese had a higher probability of acquiring HTN by more than five-fold, in comparison to individuals with normal weight. Earlier studies have determined this as a common occurrence [6,9,10,25,26,31,33]. Obesity-related HTN is thought to be associated with activation of the sympathetic nervous system, intra-abdominal and intravascular fat, sodium retention, increased renal reabsorption, and the renin–angiotensin system [34].

The present study also showed that the risk of having HTN was greater among bank employees with very high stress scores. It is obvious that bank jobs can lead to a sedentary lifestyle and variable levels of stress. A sedentary lifestyle increases atherosclerosis which, in turn, elevates the risk of HTN. Moreover, stress increases BP through producing a hormone that has an impact on vasoconstriction [26]. This finding is consistent with those of other studies in Ethiopia [25], the U.S. [35], and Australia [36].

In the current study, continuous walking for 10 min ≥ 7 times/week was observed to independently and significantly decrease the risk of hypertension among bank employees. A meta-analysis of randomized control trials (RCTs) including 5763 participants found, with moderately certain evidence, that walking decreases at least SBP for all ages and may also decrease DBP [37]. Thus, lifestyle modification practices are necessary to reduce the risk of hypertension among this high-risk group.

Consistent with the findings reported in the literature, the risk that a bank worker would have HTN increased with age [25,26]. In the univariate analysis, this trend was apparent; however, it was not clearly established in the multivariable analysis. The results obtained from other studies conducted in Ethiopia [30], Malaysia [38], and Hungary [39] support the existence of a significant relationship between aging and HTN. The discrepancy between our result and others could be attributed to the fact that the majority of our studied participants were under 40 years old, generally being younger than those in the other studies.

In the current study, sex was not associated with HTN, as the prevalence of this medical condition was equally distributed between male and female bankers. Other studies have claimed the same finding [5,6,32].

Finally, this study has some limitations. First, due to its cross-sectional design, the causal relations between the associated factors and HTN could not be established. Second, the study was conducted in one region of Saudi Arabia (i.e., the Qassim region), which may limit the generalizability of the study results among bank employees in other regions of Saudi Arabia. Further research is recommended to evaluate the prevalence of hypertension among bank employees in other regions in Saudi Arabia and to detect the causal mechanisms between hypertension and its associated factors. Moreover, the duration of employment should be considered. Despite its limitations, this study focuses on a critical topic concerning the group of bankers at high risk for HTN and provides insights in an attempt to fill the research gap regarding this neglected issue in the region. The survey aimed to identify key HTN-associated factors that could be addressed through behavioral change interventions for this occupationally exposed group.

## 5. Conclusions

As shown in this study, a significant number of bank employees had HTN. Smoking, obesity, diabetes mellitus, a positive family history of HTN, and having a very high stress level were positively associated with HTN. Meanwhile, continuous walking practice can protect against high blood pressure. Thus, it is critical to screen bank employees frequently for early detection of the condition, combined with advice for weight reduction and lifestyle modification intervention programs, in order to reduce high BP. Further longitudinal research is needed to assess the risk of HTN associated with such occupational exposure in depth.

## Figures and Tables

**Table 1:** Bank employees’ sociodemographic characteristics and HTN status.

Variables		HTN Status		*p*-Value
	Total (n = 342) N	HTN (n = 99) n (%)	Non-HTN (n = 243) n (%)	
Age group (in years) ○ 20–29 ○ 30–39 ○ 40–49 ○ ≥50	87 169 77 9	18 (20.7) 48 (28.4) 27 (35.1) 6 (66.7)	69 (79.3) 121 (71.6) 50 (64.9) 3 (33.3)	**0.015**
Sex ○ Male ○ Female	204 138	59 (28.9) 40 (29.0)	145 (71.1) 98 (71.0)	0.990
Marital status ○ Unmarried ○ Married	128 214	31 (24.2) 68 (31.8)	97 (75.8) 146 (68.2)	0.136
Educational level ○ Secondary school ○ Bachelor’s degree	119 223	36 (30.3) 63 (28.3)	83 (69.8) 160 (71.8)	0.698
Family members ○ <5 ○ 5–10 ○ >10	194 122 26	63 (32.5) 30 (24.6) 6 (23.1)	131 (67.5) 92 (75.4) 20 (76.9)	0.255
Housing ○ Rent ○ Own	111 231	36 (32.4) 63 (27.3)	75 (67.6) 168 (72.7)	0.325

*p*-values for chi-square test were provided, (bold *p*-value indicates statistical significance).

**Table 2:** Univariate analysis of studied risk factors associated with HTN among bank employees.

Variables	HTN Status		*p*-Value
	HTN (n = 99) n (%)	Non-HTN (n = 243) n (%)	
Smoking ○ No ○ Yes	56 (25.1) 43 (36.1)	167 (74.9) 76 (63.9)	**0.032**
Drinking coffee ○ <7 servings/week ○ ≥7 servings/week	23 (23.5) 76 (31.2)	75 (76.5) 168 (68.8)	0.157
Main diet ○ Breakfast ○ Lunch ○ Dinner ○ Snacks	6 (20.7) 33 (34.0) 53 (27.5) 7 (30.4)	23 (79.3) 64 (66.0) 140 (72.5) 16 (69.6)	0.492
Eating vegetables and fruits ○ <7 servings/week ○ ≥7 servings/week	88 (28.3) 11 (35.5)	223 (71.7) 20 (64.5)	0.400
Consuming fatty food ○ <7 servings/week ○ ≥7 servings/week	72 (27.6) 27 (33.3)	189 (72.4) 54 (66.7)	0.319
Adding salt while eating food ○ No ○ Yes	70 (27.6) 29 (32.9)	184 (72.4) 59 (67.1)	0.336
Number of days doing moderate physical activity ○ None ○ 1–2 days/week ○ 3–4 days/week ○ 5–6 days/week ○ Daily	39 (34.5) 18 (22.2) 12 (21.4) 15 (44.1) 15 (25.9)	74 (65.5) 63 (77.8) 44 (78.6) 19 (55.9) 43 (74.1)	0.059
Walking for 10 min continuously ○ <7/week ○ ≥7/week	86 (32.7) 13 (16.5)	177 (67.3) 66 (83.5)	**0.005**
Means of traveling from one place to another ○ On foot ○ By car	4 (40) 95 (28.6)	6 (60) 237 (71.4)	0.434
Family history of HTN ○ No ○ Yes	25 (17.1) 74 (37.8)	121 (82.9) 122 (62.2)	**<0.001**
Diabetes mellitus ○ No ○ Yes	84 (26.3) 15 (65.2)	235 (73.7) 8 (34.8)	**<0.001**
Body mass index (BMI) (kg/m^2^) ○ <25 ○ 25–30 ○ >30	23 (17.6) 28 (22.6) 48 (55.2)	108 (82.4) 96 (77.4) 39 (44.8)	**<0.001**
Perceived stress level ○ Low ○ Average ○ High ○ Very high	6 (14.6) 14 (23.7) 38 (28.6) 41 (37.6)	35 (85.4) 45 (76.3) 95 (71.4) 68 (62.4)	**0.031**

*p*-values for chi-square test were provided, (bold *p*-value indicates statistical significance).

**Table 3:** Simple and multiple logistic regression analysis of factors associated with HTN among bank employees.

Variable	Crude Odds Ratio (95% CI)	*p*-Value	Adjusted Odds Ratio (95% CI)	*p*-Value
Age group (in years)				
○ 20–29	Reference		Reference	
○ 30–39	1.52 (0.82–2.82)	0.183	0.97 (0.43–2.16)	0.935
○ 40–49	2.07 (1.03–4.16)	0.041	0.93 (0.35–2.44)	0.876
○ ≥50	7.67 (1.75–33.67)	0.007	4.12 (0.60–28.40)	0.150
Marital status				
○ Unmarried	Reference		Reference	
○ Married	1.46 (0.89–2.39)	0.137	1.26 (0.64–2.49)	0.511
Smoking				
○ No	Reference		Reference	
○ Yes	1.69 (1.04–2.73)	0.033	1.82 (1.02–3.25)	**0.043**
Drinking coffee				
○ <7 servings/week	Reference		Reference	
○ ≥7 servings/week	1.48 (0.86–2.53)	0.158	1.46 (0.0.76–2.79)	0.251
Number of days doing moderate physical activity				
○ None	Reference		Reference	
○ 1–2 days/week	0.54 (0.28–1.04)	0.066	0.91 (0.42–1.97)	0.810
○ 3–4 days/week	0.52 (0.25–1.09)	0.084	0.91 (0.38–2.15)	0.829
○ 5–6 days/week	1.50 (0.69–3.27)	0.310	1.82 (0.70–4.77)	0.220
○ Daily	0.66 (0.33–1.34)	0.251	1.28 (0.54–3.03)	0.568
Walking for 10 min continuously				
○ <7/week	Reference		Reference	
○ ≥7/week	0.41 (0.21–0.78)	0.006	0.28 (0.12–0.64)	**0.002**
Family history of HTN				
○ No	Reference		Reference	
○ Yes	2.94 (1.75–4.93)	<0.001	2.48 (1.36–4.51)	**0.003**
Diabetes mellitus				
○ No	Reference		Reference	
○ Yes	5.25 (2.15–12.82)	<0.001	5.14 (1.60–16.54)	**0.006**
BMI (kg/m^2^)				
○ <25	Reference		Reference	
○ 25–30	1.37 (0.74–2.54)	0.317	1.22 (0.61–2.44)	0.578
○ >30	5.78 (3.12–10.71)	<0.001	5.49 (2.75–10.96)	**<0.001**
Perceived stress level				
○ Low	Reference		Reference	
○ Average	1.81 (0.63–5.20)	0.267	1.99 (0.57–6.78)	0.269
○ High	2.33 (0.91–5.99)	0.079	1.89 (0.61–5.85)	0.270
○ Very high	3.52 (1.36–9.08)	0.009	3.24 (1.04–10.11)	**0.043**

Bold *p*-value indicates statistical significance.

## Data Availability

The data presented in this study are available on request made to the corresponding author.

## Supplementary Materials

The following supporting information can be downloaded at: https://www.mdpi.com/article/10.3390/healthcare13020134/s1. Table S1: Informed consent was obtained from each bank employee after they were informed about the study’s aim.
